# Serum homocysteine level is an independent risk factor for 1-year stroke recurrence in patients with acute ischemic stroke and H-type hypertension: results from the Xi'an stroke registry study of China

**DOI:** 10.3389/fneur.2023.1161318

**Published:** 2023-04-18

**Authors:** Dandan Zhang, Zhongzhong Liu, Weiyan Guo, Qingli Lu, Huan Zhang, Zhen Lei, Pei Liu, Congli Huang, Jing Wang, Qiaoqiao Chang, Xuemei Lin, Fang Wang, Songdi Wu

**Affiliations:** ^1^Department of Neurology, Xi'an No.1 Hospital, The First Affiliated Hospital of Northwest University, Xi'an, China; ^2^Xi'an Key Laboratory for Innovation and Translation of Neuroimmunological Diseases, Xi'an No.1 Hospital, The First Affiliated Hospital of Northwest University, Xi'an, China; ^3^Department of Epidemiology and Biostatistics, School of Public Health of Xi'an Jiaotong University Health Science Center, Xi'an, China; ^4^College of Life Science, Northwest University, Xi'an, China; ^5^Department of Traditional Chinese Medicine, Xi'an No.1 Hospital, The First Affiliated Hospital of Northwest University, Xi'an, China

**Keywords:** serum, homocysteine, acute ischemic stroke, H-type hypertension, prognosis

## Abstract

**Background:**

H-type hypertension has a high prevalence in China. However, the association of serum homocysteine levels with 1-year stroke recurrence in patients with acute ischemic stroke (AIS) and H-type hypertension has not been studied.

**Methods:**

A prospective cohort study of patients with AIS admitted to hospitals between January and December 2015 in Xi'an, China, was conducted. Serum homocysteine levels, demographic data, and other relevant information were collected from all patients upon admission. Stroke recurrences were routinely tracked at 1, 3, 6, and 12 months after discharge. The blood homocysteine level was studied as a continuous variable and tertiles (T1–T3). A multivariable Cox proportional hazard model and a two-piecewise linear regression model were utilized to evaluate the association and ascertain the threshold effect regarding the serum homocysteine level and 1-year stroke recurrence in patients with AIS and H-type hypertension.

**Results:**

Overall, 951 patients with AIS and H-type hypertension were enrolled, of whom 61.1% were male. After adjusting for confounders, patients in T3 had a significantly increased risk of recurrent stroke within 1 year, compared with those in T1 as the reference (hazard ratio = 2.24, 95% confidence interval: 1.01–4.97, *p* = 0.047). Curve fitting showed that serum homocysteine levels were positively curvilinearly correlated with 1-year stroke recurrence. Threshold effect analysis showed that an optimal threshold of serum homocysteine level <25 μmol/L was effective in reducing the risk of 1-year stroke recurrence in patients with AIS and H-type hypertension. Elevated homocysteine levels in patients with severe neurological deficits on admission significantly increased the risk of 1-year stroke recurrence (*p* for interaction = 0.041).

**Conclusions:**

In patients with AIS and H-type hypertension, the serum homocysteine level was an independent risk factor for 1-year stroke recurrence. A serum homocysteine level of ≥25 μmol/L significantly increased the risk of 1-year stroke recurrence. These findings can inform the creation of a more precise homocysteine reference range for the prevention and treatment of 1-year stroke recurrence in patients with AIS and H-type hypertension and provide a theoretical foundation for the individualized prevention and treatment of stroke recurrence.

## 1. Introduction

Stroke is the second leading cause of mortality worldwide and the primary cause of disability and mortality in China (1–3). In 2020, ischemic stroke constituted 15.5 million (86.8%) of all incident stroke cases in the Chinese population aged ≥40 years (4). Hypertension is one of the leading risk factors for stroke (1–4). Previous studies have revealed a closer relationship between hypertension and stroke in Asian populations, especially among the Chinese, than that in the European, American, Australian, and New Zealand populations (5–7).

H-type hypertension is used to describe hypertensive patients with hyperhomocysteinemia (HHcy, homocysteine levels ≥10 μmol/L) (7, 8). H-type hypertension is a major disease in China, with a prevalence of 29.5% in the Chinese hypertensive population (9). Furthermore, H-type hypertension is an independent predictor of stroke (9), increases the risk of early cognitive impairment (10), is a dangerous predictor of small vessel disease of the brain (11), and may lead to a higher rate of stroke recurrence (12, 13). Most previous studies have discussed the influence of H-type hypertension on stroke occurrence and prognosis. The correlation between the serum homocysteine level and 1-year stroke recurrence in patients with acute ischemic stroke (AIS) and H-type hypertension has, however, hardly been studied. In addition, the optimal threshold range for serum homocysteine levels to effectively reduce the risk of 1-year stroke recurrence in patients with AIS and H-type hypertension has been poorly studied.

Based on the results of the Xi'an Stroke Registry Database, we investigated the association of the serum homocysteine level with 1-year stroke recurrence in patients with AIS and H-type hypertension, aiming to provide a more precise homocysteine reference range for the prevention and treatment of 1-year stroke recurrence in patients with AIS and H-type hypertension and a scientific theoretical foundation for customized stroke recurrence prevention and therapy.

## 2. Materials and methods

### 2.1. Study population

**Figure 1:**
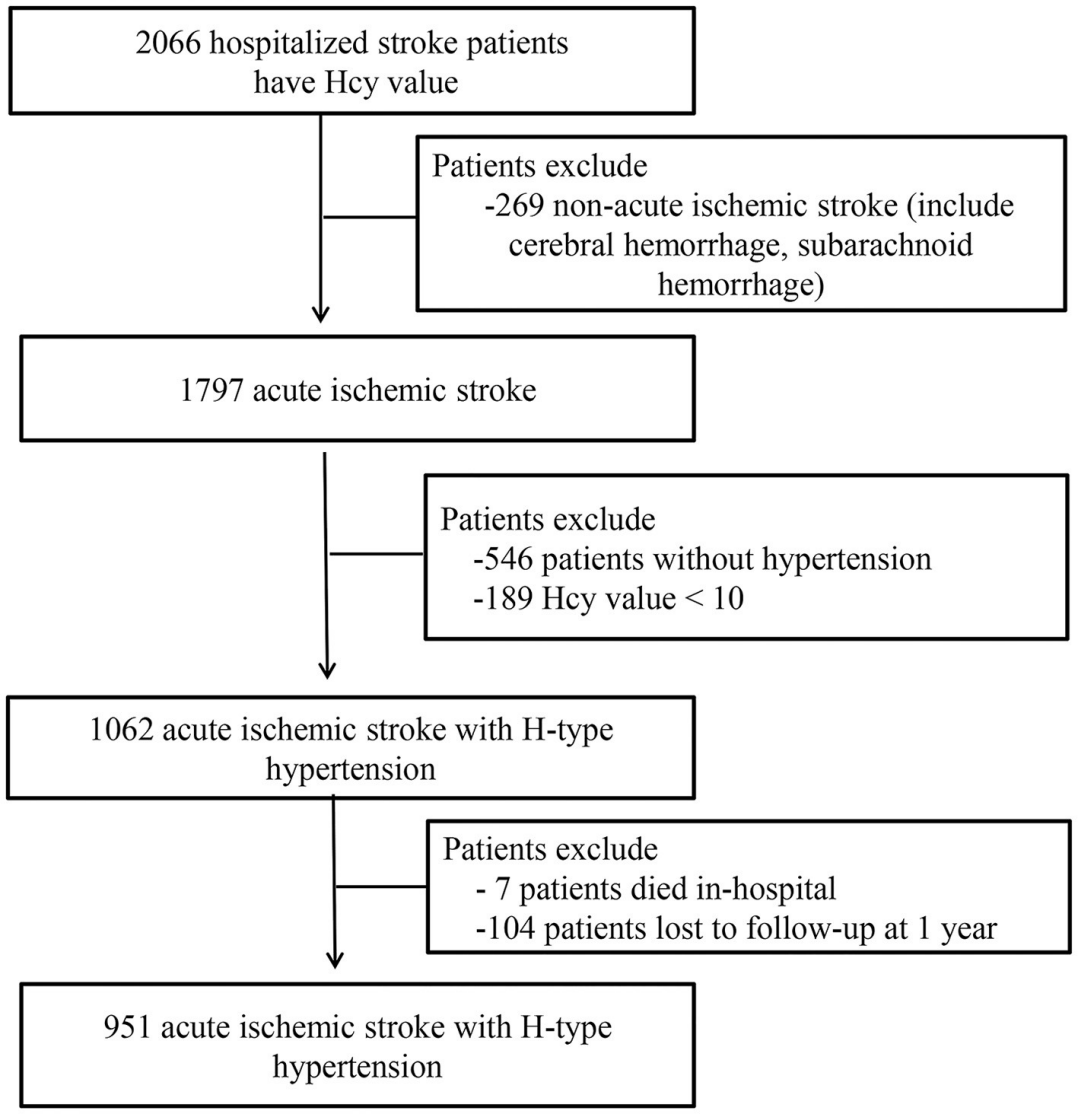
Screening flow chart for enrolled patients. HHcy, homocysteine.

This study included patients with any stroke subtype admitted to four first-class tertiary hospitals between January and December 2015 in Xi'an, China. A total of 2,066 patients with stroke received comprehensive medical examinations at the initial phase of the study. After the symptom onset, follow-ups were performed at 1, 3, 6, and 12 months. Among these patients, 951 patients with AIS and H-type hypertension were finally included in the analysis after excluding those without AIS (*n* = 269) and hypertension (*n* = 546), those with a homocysteine value <10 μmol/L (*n* = 189), those who died in hospital (*n* = 7), and those lost to follow-up at 1 year (*n* = 104). The detailed screening process and research flow chart are shown in Figure 1. All the participating hospitals used consistent diagnostic criteria.

### 2.2. Standard protocol approvals and patient consent

The Declaration of Helsinki was followed when conducting the present study. The Academic Committee of Xi'an No. 1 hospital and the ethics committees of all the participating hospitals approved the study (Approval No. 2014 [5], Registration number: ChiCTR-EOC-17012190). All the patients provided written and oral informed consent.

### 2.3. Baseline data collection

Using information from the Xi'an Stroke Registry, we conducted this multicenter observational cohort study (14). Baseline data were gathered, including sociodemographic data, medical history, hospital admission evaluation, and important laboratory tests (Table 1). In addition to hyperhomocysteinemia, the relevant criteria for medical history, risk factors, and definitions were consistent with those used in the Chinese Intracranial Atherosclerosis Study (15). The serum homocysteine level was analyzed as both a continuous and categorical variable (tertiles, T1–T3). The serum homocysteine levels were classified as follows, from low to high: T1: <15.8 μmol/L, T2:15.8–24.6 μmol/L, and T3: ≥24.7 μmol/L.

**Table 1:** Baseline characteristics by homocysteine tertiles (T1–T3) in patients with AIS and H-type hypertension.

**Variables**	**Overall *n* = 951**	**Homocysteine tertiles**			***P*** **-value**
		**T1**, ***n*** = **317**	**T2**, ***n*** = **317**	**T3**, ***n*** = **317**	
**Demographic information**					
Age (years)	65.5 ± 11.7	65.5 ± 12.2	66.6 ± 11.7	64.5 ± 11.0	0.074
Sex, *n* (%)					<0.001
Male	581 (61.1)	167 (52.7)	203 (64)	211 (66.6)	
Female	370 (38.9)	150 (47.3)	114 (36)	106 (33.4)	
Education level, *n* (%)					0.708
Elementary or below	436 (45.8)	146 (46.1)	145 (45.7)	145 (45.7)	
Middle school	200 (21.0)	65 (20.5)	74 (23.3)	61 (19.2)	
High school or above	315 (33.1)	106 (33.4)	98 (30.9)	111 (35)	
**Cerebrovascular risk factors**					
Smoking, *n* (%)					<0.001
Never smoking	538 (56.6)	210 (66.2)	177 (55.8)	151 (47.6)	
Smoking cessation	178 (18.7)	50 (15.8)	67 (21.1)	61 (19.2)	
Current smoking	235 (24.7)	57 (18)	73 (23)	105 (33.1)	
Drinking, *n* (%)					0.32
No	717 (75.4)	248 (78.2)	237 (74.8)	232 (73.2)	
Yes	234 (24.6)	69 (21.8)	80 (25.2)	85 (26.8)	
**Examination on admission**					
BMI (kg/m^2^)	24.1 ± 3.7	23.8 ± 3.5	24.4 ± 4.0	24.1 ± 3.7	0.113
SBP on admission (mmHg)	150.4 ± 22.5	151.5 ± 22.2	150.7 ± 21.8	148.9 ± 23.5	0.324
DBP on admission (mmHg)	88.3 ± 13.3	87.5 ± 12.5	87.8 ± 13.2	89.8 ± 14.1	0.050
Heart rate (times per minute)	74.7 ± 10.6	75.6 ± 11.2	74.1 ± 10.5	74.5 ± 10.0	0.209
NIHSS score on admission, *n* (%)					0.003
< 4	444 (46.7)	170 (53.6)	147 (46.4)	127 (40.1)	
4–14	434 (45.6)	122 (38.5)	150 (47.3)	162 (51.1)	
>14	73 (7.7)	25 (7.9)	20 (6.3)	28 (8.8)	
Pneumonia, *n* (%)					0.566
No	899 (94.5)	303 (95.6)	299 (94.3)	297 (93.7)	
Yes	52 (5.5)	14 (4.4)	18 (5.7)	20 (6.3)	
**Previous medical history**					
Prior stroke, *n* (%)					0.651
No	631 (66.4)	204 (64.4)	214 (67.5)	213 (67.2)	
Yes	320 (33.6)	113 (35.6)	103 (32.5)	104 (32.8)	
Diabetes mellitus, *n* (%)					<0.001
No	708 (74.4)	211 (66.6)	241 (76)	256 (80.8)	
Yes	243 (25.6)	106 (33.4)	76 (24)	61 (19.2)	
Atrial fibrillation, *n* (%)					0.370
No	878 (92.3)	291 (91.8)	289 (91.2)	298 (94)	
Yes	73 (7.7)	26 (8.2)	28 (8.8)	19 (6)	
**Laboratory findings**					
Total cholesterol (mmol/L)	4.4 ± 1.1	4.4 ± 1.1	4.4 ± 1.2	4.4 ± 1.0	0.900
Triglycerides (mmol/L)	1.7 ± 1.5	1.7 ± 1.2	1.7 ± 1.5	1.8 ± 1.6	0.675
HDL-cholesterol (mmol/L)	1.1 ± 0.3	1.2 ± 0.3	1.1 ± 0.3	1.1 ± 0.3	0.021
LDL-cholesterol (mmol/L)	2.6 ± 0.8	2.6 ± 0.9	2.6 ± 0.9	2.6 ± 0.8	0.770
FPG (mmol/L)	6.1 ± 2.5	6.4 ± 2.8	6.0 ± 2.6	5.7 ± 2.1	0.004
Alanine aminotransferase (U/L)	24.2 ± 17.8	24.3 ± 18.1	24.2 ± 17.0	24.1 ± 18.2	0.992
Aspartate aminotransferase (U/L)	25.4 ± 15.7	24.8 ± 11.3	25.4 ± 13.7	26.1 ± 20.6	0.568
Alkaline phosphatase (U/L)	80.9 ± 27.4	80.1 ± 25.9	81.9 ± 30.1	80.8 ± 26.1	0.697
Serum creatinine (μmol/L)	77.9 ± 37.5	73.0 ± 39.0	76.8 ± 26.9	83.8 ± 43.8	0.001
Blood urea nitrogen (mmol/L)	5.3 ± 1.9	5.2 ± 1.8	5.3 ± 1.9	5.4 ± 2.0	0.370
Uric acid (μmol/L)	291.6 ± 98.9	282.5 ± 96.0	292.5 ± 106.0	299.4 ± 94.0	0.108
Leukocyte count ( × 10^9^/L)	6.9 ± 2.5	6.8 ± 2.5	6.9 ± 2.6	7.1 ± 2.5	0.425
Platelet count (× 10^9^/L)	186.1 ± 59.5	191.9 ± 61.6	184.7 ± 57.7	181.7 ± 58.9	0.087
Homocysteine (μmol/L)	24.2 ± 14.7	12.8 ± 1.7	19.5 ± 2.5	40.2 ± 15.3	<0.001

Data presented are mean ± SD, median (Q1–Q3), or N (%).

BMI, body mass index; SBP, systolic blood pressure; DBP, diastolic blood pressure; NIHSS, national institutes of health stroke scale; HDL-cholesterol, high-density lipoprotein cholesterol; LDL-cholesterol, low-density lipoprotein cholesterol; FPG, fasting venous plasma glucose; T, tertiles. T1: < 15.8 μmol/L, T2:15.8–24.6 μmol/L, T3: ≥24.7μmol/L.

### 2.4. Measurements and outcomes

Within 24 h of admission, fasting venous blood samples were collected, and the serum homocysteine levels in unfrozen samples were tested by high-performance liquid chromatography according to the manual (12). Smoking was considered the smoking of at least one cigarette each day before stroke onset for more than or an accumulation of 6 months; smoking cessation was defined as having previously met the definition of smoking but not smoking for six consecutive months prior to the stroke. Drinking was defined as drinking ~50 mL of alcohol per week prior to stroke onset. On admission to the hospital, stroke was categorized according to the National Institutes of Health Stroke Scale (NIHSS) score: a mild deficit (<4), a moderate deficit (4–14), and a severe deficit (>14). Hypertension was defined as a history of hypertension or two instances of a resting systolic blood pressure ≥140 mmHg and/or diastolic blood pressure ≥90 mmHg. H-type hypertension was defined as hypertension with HHcy and homocysteine levels ≥10 μmol/L. According to previous studies, the cut-off value for the homocysteine level was selected as ≥10 μmol/L (7, 16–18). This study's endpoint event, a new acute stroke event at the 1-year follow-up (including cerebral infarction, cerebral hemorrhage, and subarachnoid hemorrhage), was defined as a 1-year stroke recurrence. A new acute stroke event was identified by an independent panel comprising four to five stroke experts from each hospital.

### 2.5. Follow-up

After AIS onset, follow-ups were performed at 1, 3, 6, and 12 months. Less than 5 days was the permissible inaccuracy in follow-up time. The trained research coordinators followed up all enrolled patients *via* face-to-face interviews or telephone calls and recorded the dates of stroke recurrence. Patients were deemed lost to follow-up if they declined to participate in the research or were unable to be reached by phone after three daily attempts for five consecutive business days.

### 2.6. Statistical analyses

Normally distributed continuous variables are presented as mean ± standard deviation, while categorical variables and deviation are expressed as frequencies and percentages. Based on the different distribution of data, the one-way analysis of variance, Chi-square test, and non-parametric test were used for comparison between the groups. When the theoretical frequency was <10, Fisher's exact test was employed. Multivariable Cox proportional hazard models were used to analyze the relationship between the homocysteine levels and 1-year stroke recurrence. Covariates were included in the adjusted model as potential confounding factors if they changed the estimates of the serum homocysteine levels at 1-year stroke recurrence by more than 10% or were significantly associated with 1-year stroke recurrence. A likelihood ratio test was used to confirm the subgroup interactions. A two-tailed value *p* < 0.05 was deemed statistically significant. All the analyses were performed with the Free Statistics software version 1.3 (www.clinicalscientists.cn, free clinical Medical Technology, Inc., Beijing, China) and R statistical software version 3.3.2 (www.R-project.org; The R Foundation).

## 3. Results

### 3.1. Baseline characteristics

A total of 951 patients (581 men, 370 women) remained in the study at the completion of the 1-year follow-up (mean age: 65.5 ± 11.7 years). The baseline clinical, biochemical, and demographic characteristics of the homocysteine tertiles (T1–T3) were compared (Table 1). Men, current smokers, and those with high NIHSS scores on admission as well as those with high serum creatinine levels had higher homocysteine levels. Furthermore, increased homocysteine levels were significantly inversely proportional to the ratios of the presence of diabetes mellitus, high-density lipoprotein cholesterol, and fasting venous plasma glucose levels. No significant differences were observed in the educational level, age, body mass index, drinking, systolic blood pressure on admission, diastolic blood pressure on admission, heart rate on admission, pneumonia, prior stroke, atrial fibrillation, triglycerides, total cholesterol, low-density lipoprotein cholesterol, alanine aminotransferase, aspartate aminotransferase, alkaline phosphatase, blood urea nitrogen, uric acid, leukocyte count, or platelet count.

### 3.2. Association between serum homocysteine levels and 1-year stroke recurrence in patients with AIS with H-type hypertension

**Figure 2:**
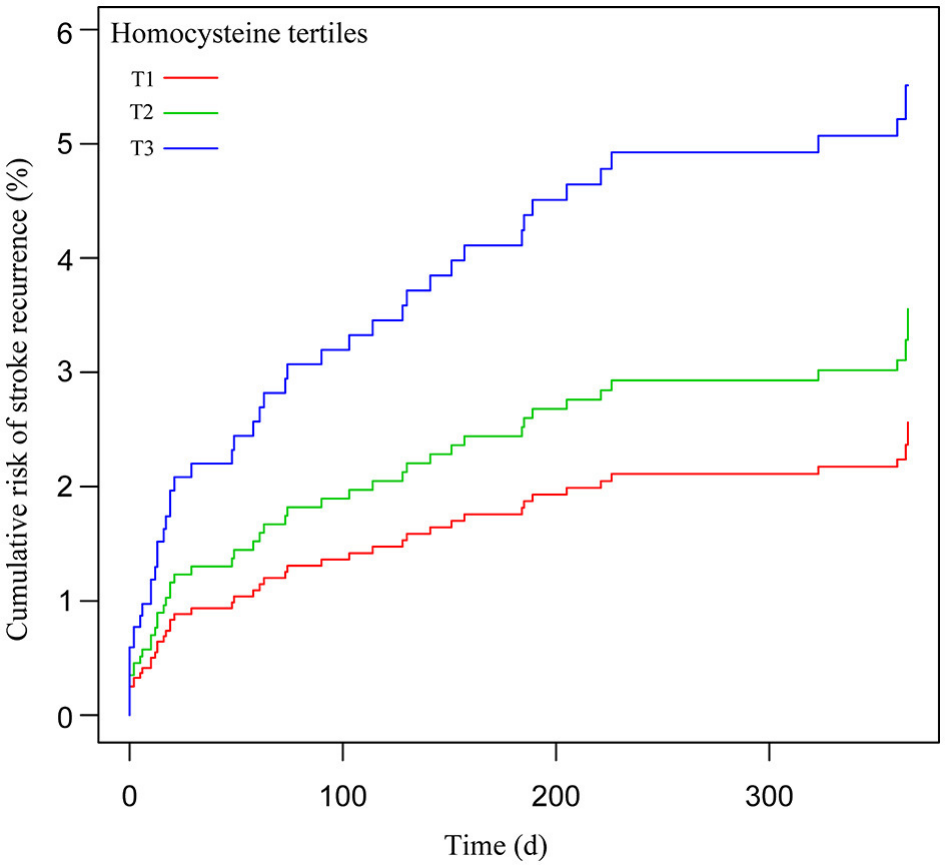
Cumulative risk of 1-year stroke recurrence after homocysteine level as tertiles categorical variables (T1–T3).

Table 2 displays the findings of the crude and multivariate-adjusted Cox regression models. First, the homocysteine level was used as a continuous variable for analysis. In the crude model, for every 5-unit (5 μmol/L) increase in homocysteine, the risk of 1-year stroke recurrence increased by 9% (hazard ratio [HR] = 1.09, 95% confidence interval [CI]: 1.02–1.17, *p* = 0.014). In the adjusted model, the risk of 1-year stroke recurrence increased by 13% (HR = 1.13, 95% CI: 1.04–1.22, *p* = 0.003). Thereafter, the homocysteine level was analyzed as a categorical variable (tertiles T1–T3). After adjustment for potential confounders, patients in T3 had a higher risk of 1-year stroke recurrence than did those in T1 as the reference tertile (HR = 2.24, 95% CI: 1.01–4.97, *p* = 0.047). Each tertile of the adjusted model showed a significant statistical difference in the trend of rising hazard according to the trend test (*p* for trend = 0.035). Furthermore, the Kaplan–Meier curve revealed that the cumulative stroke recurrence rate in 1 year in T3 was significantly higher than that in T1 (Figure 2).

**Table 2:** Multivariate cox regression analysis of the homocysteine level and 1-year stroke recurrence in patients with AIS and H-type hypertension.

**Variable**	**Overall, *n***	**Event, *n* (%)**	**Crude model HR (95%CI)**	***P-*** **value**	**Adjusted model HR (95%CI)**	***P-*** **value**
Homocysteine, per 5 units increase	951	56 (5.9)	1.09 (1.02~1.17)	0.014	1.13 (1.04~1.22)	0.003
**Homocysteine tertiles**						
T1	317	13 (4.1)	Ref.		Ref.	
T2	317	17 (5.4)	1.33 (0.64~2.73)	0.441	1.39 (0.59~3.25)	0.454
T3	317	26 (8.2)	2.08 (1.07~4.04)	0.032	2.24 (1.01~4.97)	0.047
*P* for trend. test				0.027		0.035

Crude adjust none; adjusted for age, sex, smoking, drinking, prior stroke, NIHSS score at admission, pneumonia, total cholesterol, triglyceride, HDL-cholesterol, LDL-cholesterol, FPG, alkaline phosphatase, leukocyte count (× 10^9^/L), BMI, diabetes mellitus, and atrial fibrillation. HR, hazard ratio; CI, confidence interval; and T, tertiles. T1: <15.8 μmol/L, T2: 15.8–24.6 μmol/L, T3: ≥24.7 μmol/L.

### 3.3. Threshold effect analysis of homocysteine levels regarding 1-year stroke recurrence in patients with AIS and H-type hypertension

**Figure 3:**
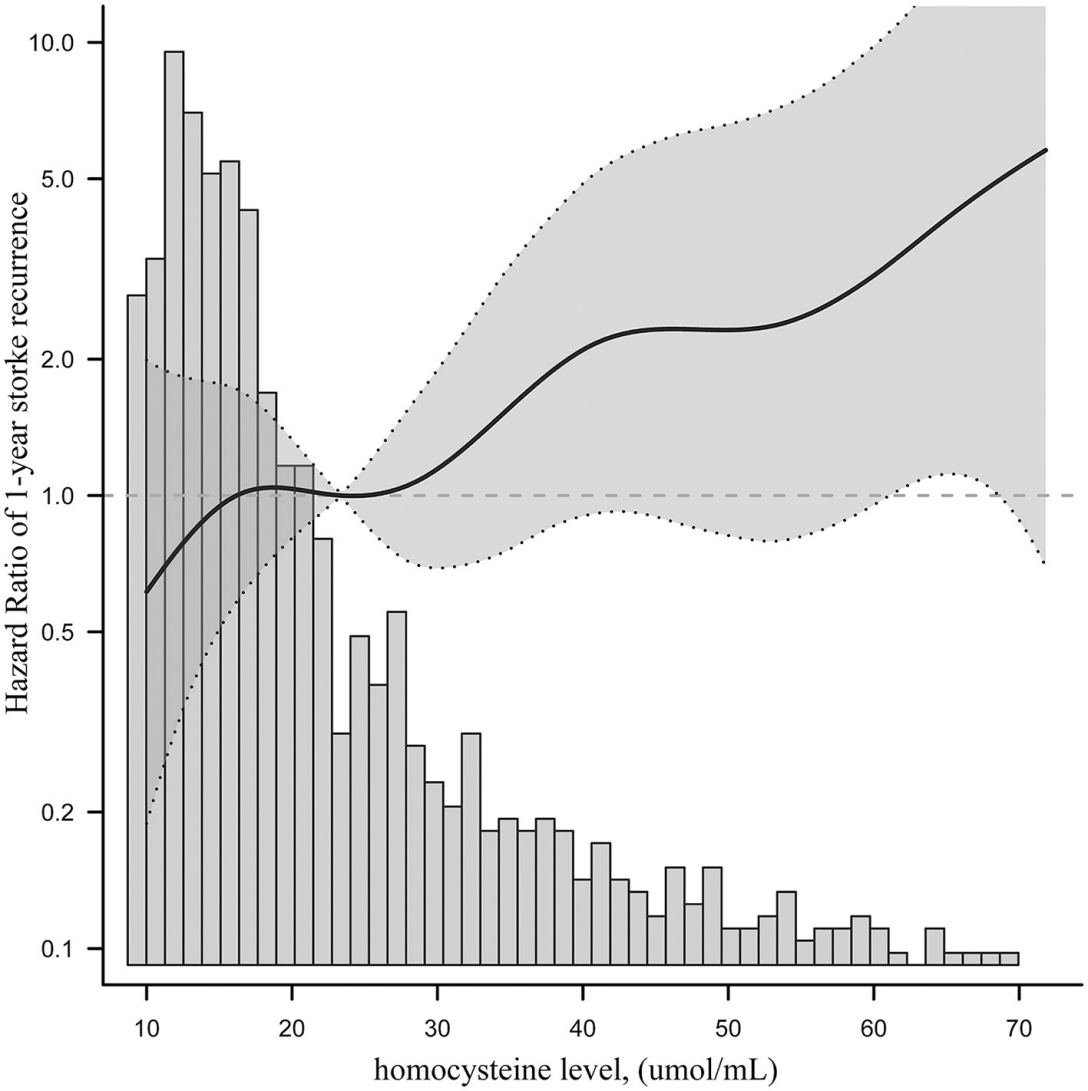
The smoothing curve fitting of serum homocysteine level and 1-year stroke recurrence.

After adjustment for potential confounders, the smoothing curve fitting revealed a curvilinear relationship, with a positive correlation between the serum homocysteine levels and 1-year stroke recurrence in patients with AIS with H-type hypertension (Figure 3). According to the two-piecewise linear multivariate Cox regression model, 25 μmol/L was the optimal homocysteine threshold value. The likelihood ratio test showed no significant statistical difference between the two linear models. However, multivariate Cox regression showed that in patients with homocysteine levels below the optimal threshold value, the homocysteine levels (per 1 μmol/L increase) were not significantly associated with the risk of 1-year stroke recurrence in AIS patients with H-type hypertension (HR = 0.99, 95% CI: 0.89–1.11, *p* = 0.935), whereas in patients with homocysteine levels above the optimal threshold value, the risk of 1-year stroke recurrence significantly increased (HR = 1.04, 95% CI: 1.01–1.07, *p* = 0.007; Table 3).

**Table 3:** Threshold effect analysis of homocysteine and 1-year stroke recurrence in patients with AIS and H-type hypertension.

**Variable**	**HR (95%CI)**	***P*** **-value**
One-line linear regression model	1.03 (1.01~1.04)	0.002
**Two-piecewise linear regression model**		
Homocysteine <25 μmol/L	0.99 (0.89~1.11)	0.935
Homocysteine ≥25 μmol/L	1.04 (1.01~1.07)	0.007
Likelihood ratio test		0.500

Adjusted for age, sex, smoking, drinking, prior stroke, NIHSS score at admission, pneumonia, total cholesterol, triglyceride, HDL-cholesterol, LDL-cholesterol, FPG, alkaline phosphatase, leukocyte count (× 10^9^/L), BMI, diabetes mellitus, and atrial fibrillation.

### 3.4. Subgroups analyses

**Figure 4:**
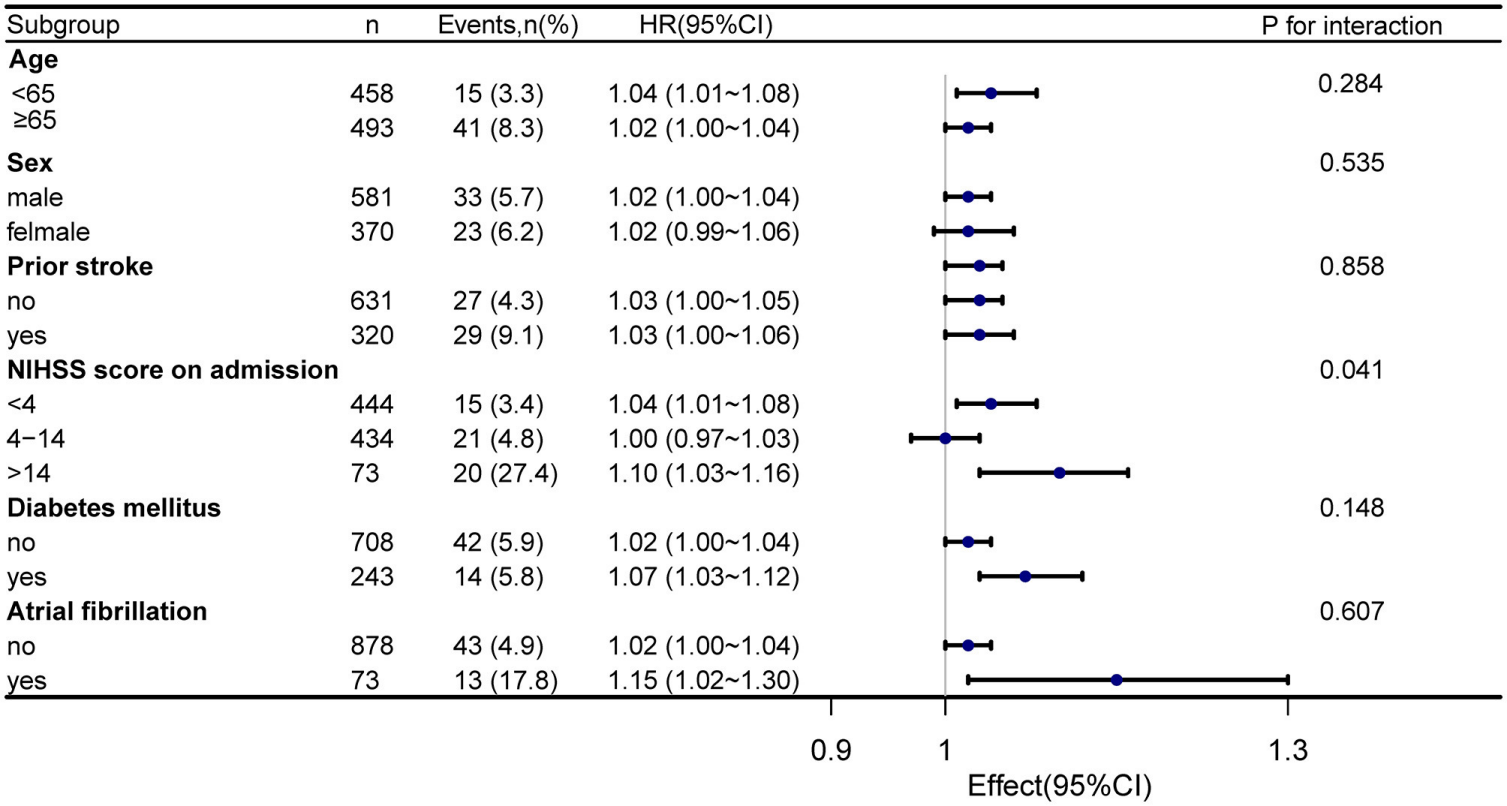
Forest map subgroup analysis of serum homocysteine level and 1-year stroke recurrence. Stratified by age, sex, prior stroke, NIHSS score on admission, diabetes mellitus, and atrial fibrillation.

Stratified and interactive analyses were performed to evaluate whether the association of serum homocysteine levels with 1-year stroke recurrence was consistent across the subgroups (Figure 4). The results revealed that the NIHSS score on admission (stratified by scores of 4 and 14) performed an interactive role in the association of serum homocysteine levels with 1-year stroke recurrence (*p* for interaction = 0.041). Increased homocysteine levels in patients with an NIHSS score of >14 on admission significantly increased the risk of 1-year stroke recurrence (HR = 1.10, 95% CI: 1.03–1.16). Although there was no significant interaction between age, sex, incidence of prior stroke, diabetes mellitus, and atrial fibrillation, the stratified analysis also found a higher risk of 1-year stroke recurrence in patients with diabetes mellitus (HR = 1.07, 95% CI: 1.03–1.12) and atrial fibrillation (HR = 1.15, 95% CI: 1.02–1.30).

## 4. Discussion

We found that in patients with AIS and H-type hypertension, serum homocysteine was considered an independent risk factor for 1-year stroke recurrence, and serum homocysteine had a positively correlated curvilinear relationship with 1-year stroke recurrence. An optimal threshold of serum homocysteine level <25 μmol/L was effective in reducing the risk of 1-year stroke recurrence in patients with AIS and H-type hypertension. Elevated homocysteine levels in patients with severe neurological deficits on admission significantly increased the risk of 1-year stroke recurrence.

A review of the literature found that H-type hypertension is a major risk factor for stroke incidence and stroke mortality (18) and may lead to poor prognosis in patients with AIS (19). However, most studies only focus on the effect of the presence or absence of H-type hypertension on stroke occurrence and outcome, and the correlation between serum homocysteine levels and stroke recurrence in patients with AIS and H-type hypertension has rarely been studied. In addition, the optimal range of serum homocysteine levels for recurrent stroke remains unclear. This multicenter prospective cohort study evaluated the association of serum homocysteine levels with 1-year stroke recurrence and provided a more accurate homocysteine reference range for the prevention and treatment of recurrent stroke in patients with AIS and H-type hypertension, based on the Xi'an Stroke Registry Database.

Previous studies have analyzed patients with AIS with and without H-type hypertension (12, 13, 18, 19). Zhang et al. (12) performed a cross-sectional observational study and revealed that recurrent ischemic stroke is associated with H-type hypertension. Li et al. (13) also conducted a study on the relationship between H-type hypertension and stroke prognosis and found that H-type hypertension might lead to a higher occurrence rate of endpoint events, especially recurrent stroke. However, even after controlling for confounding factors, the results did not fully reflect the independent role of serum homocysteine levels in stroke recurrence in patients with AIS and H-type hypertension. When the homocysteine level was analyzed as a continuous variable after correcting for potential confounding variables, our study demonstrated that every 5 μmol/L rise in the homocysteine level raised the risk of 1-year stroke recurrence by 13%. This suggests that a higher serum homocysteine level is associated with an increased risk of 1-year stroke recurrence in patients with AIS and H-type hypertension.

In addition, when homocysteine was used as a categorical variable (tertiles), the risk was 1.24 times higher in T3 compared to that in T1, which was considered a reference for patients with AIS and H-type hypertension. However, no significant difference in the risk between T2 and T1 was observed (Table 2). These findings suggest the possibility of a threshold effect regarding the role of homocysteine levels in stroke recurrence. In other words, when the homocysteine level reaches a certain threshold, it may obviously cause the recurrence of stroke in patients with AIS and H-type hypertension. To date, the optimal range of the serum homocysteine level for 1-year stroke recurrence has not been clarified, especially in patients with AIS and H-type hypertension. To clarify this correlation, we investigated the threshold effect and dose-reaction association of homocysteine levels with 1-year stroke recurrence in a large multicenter prospective cohort study. After controlling for potential confounders, a positive correlation between the serum homocysteine level and 1-year stroke recurrence was found in patients with AIS and H-type hypertension (Figure 3). The threshold effect analysis revealed that when the serum homocysteine level was ≥25 μmol/L in patients with AIS and H-type hypertension, higher serum homocysteine levels were associated with a greater risk of 1-year stroke recurrence. However, when the serum homocysteine level was <25 μmol/L, the risk of 1-year stroke recurrence was not significantly correlated with homocysteine levels (Table 3). Clinicians should focus on homocysteine levels in patients with AIS and H-type hypertension, and intervention when serum levels are above 25 μmol/L may effectively reduce the risk of 1-year stroke recurrence.

Due to the large population, wide geographical area, and differences in dietary habits, homocysteine levels are affected to varying degrees in China, thus resulting in large differences in survey results (20). According to epidemiological data, the prevalence of H-type hypertension in China is higher in the inland, western, and northern regions (21). The Xi'an district is located in the northwest of the Chinese inland, which is a very representative area. Folic acid reduces homocysteine levels (22–24). However, folic acid supplementation or fortification is not widely used in China (25). If the range of serum homocysteine levels affecting stroke recurrence can be defined and homocysteine levels can be reduced to the optimal range, a theoretical basis can be developed for formulating a treatment plan that matches the relevant population characteristics and aids in the precise treatment of patients with AIS and H-type hypertension. We found that a serum homocysteine level of ≥25 μmol/L significantly increased the risk of 1-year stroke recurrence. This provides a scientific basis for the prevention and treatment of stroke recurrence in patients with AIS and H-type hypertension, especially in the Xi 'an area.

The subgroup analysis found that the NIHSS score on admission was an interaction factor between serum homocysteine and stroke recurrence, and patients with more severe neurological impairment were more likely to experience stroke recurrence. The NIHSS score is a direct indication of the degree of neurologic deficit and has high accuracy in assessing prognosis (26, 27). There is presently no particular therapy for stroke-induced cerebral functional impairment, and the prognosis for patients with severe neurological impairment is generally poor. We also observed no significant interaction between serum homocysteine levels and 1-year stroke recurrence in patients with diabetes mellitus and atrial fibrillation. However, the stratified analysis also found a higher risk of 1-year stroke recurrence in patients with diabetes mellitus (HR = 1.07, 95% CI: 1.03–1.12) and atrial fibrillation (HR = 1.15, 95% CI: 1.02–1.30). Previous studies have indicated that stroke related to atrial fibrillation has not only a high incidence of severe disability but also a high risk of recurrence (28, 29). In addition, Zhang et al. (30) observed that diabetes mellitus is an independent risk factor for recurrent stroke in ischemic stroke patients. In summary, more emphasis should be placed on the consequences of homocysteine changes and recurrent stroke in patients with severe neurological dysfunction, atrial fibrillation, or diabetes.

The underlying mechanism of the association of a higher homocysteine level with stroke recurrence in patients with AIS and H-type hypertension remains unclear. HHcy induces complex changes in vascular walls, including oxidative stress, endothelial cell damage, altered lipid metabolism, and the promotion of thrombosis (31, 32). Studies have also revealed that HHcy stimulates angiotensin-converting enzymes by decreasing the synthesis of endogenous hydrogen sulfide in the body, and the subsequently produced angiotensin II acts on the corresponding receptors, resulting in a sequence of pathological conditions, such as elevated blood pressure and angiogenesis (33–35). Xu et al. discovered that serum homocysteine levels are negatively correlated with the proportion of CD4+ T cells. The decrease in total CD4+ T cells further reduces the number of blood pressure-protective Treg cells and leads to a relative increase in pro-inflammatory cytokines, thereby aggravating hypertension and damage to target organs (36). Additionally, our research found that serum homocysteine levels ≥25 μmol/L in patients with AIS and H-type hypertension were significantly associated with an increased risk of 1-year stroke recurrence. This finding suggests that abnormally elevated serum homocysteine levels may adversely affect the risk of recurrent stroke. In addition, addressing abnormal serum homocysteine levels is a potential therapeutic target for improving recurrent stroke risk. However, further research is needed to confirm this finding.

This research has several limitations. First, the homocysteine levels were only assessed once within 24 h after stroke onset, and no changes in the homocysteine levels or blood pressure were observed throughout the duration of the long-term clinical follow-up. Second, no records of diet or drugs that affect the homocysteine metabolism were available. Third, data on homocysteine-related genes, such as MTHFR and MTRR, were not available. In addition, the four hospitals included in this research were all local third-class hospitals, and there were no second- or first-class hospitals; thus, selection bias is possible.

## 5. Conclusion

Our study revealed that the serum homocysteine level is an independent risk factor for 1-year stroke recurrence in patients with AIS and H-type hypertension. A serum homocysteine level of ≥25 μmol/L could significantly increase the risk of 1-year stroke recurrence. Our findings suggest that clinicians should be concerned about elevated homocysteine levels in patients with AIS and H-type hypertension, and early attention and the necessary intervention may help reduce the risk of 1-year stroke recurrence.

## Data availability statement

The original contributions presented in the study are included in the article/supplementary material, further inquiries can be directed to the corresponding author.

## Ethics statement

The studies involving human participants were reviewed and approved by Ethics Committee at Xi'an No.1 Hospital. The patients/participants provided their written informed consent to participate in this study.

## Author contributions

SW had full access to all of the data in the study and takes responsibility for the integrity of the data, the accuracy of the data analysis, and revised the manuscript for important intellectual content. DZ and ZLi planned and designed the study, contributed to the data cleaning and statistical analysis, and wrote the manuscript. WG, QL, HZ, ZLe, PL, CH, JW, QC, XL, and FW contributed to the follow-up of the patients and recorded the data at each stage. All authors read and approved the final version of the manuscript.
